# Using Magnetic Resonance Imaging to Evaluate Dendritic Cell-Based Vaccination

**DOI:** 10.1371/journal.pone.0065318

**Published:** 2013-05-29

**Authors:** Peter M. Ferguson, Angela Slocombe, Richard D. Tilley, Ian F. Hermans

**Affiliations:** 1 Malaghan Institute of Medical Research, Wellington, New Zealand; 2 Department of Radiology, Wellington Hospital, Wellington, New Zealand; 3 School of Chemical and Physical Sciences, Victoria University of Wellington, Wellington, New Zealand; Glaxo Smith Kline, Denmark

## Abstract

Cancer immunotherapy with antigen-loaded dendritic cell-based vaccines can induce clinical responses in some patients, but further optimization is required to unlock the full potential of this strategy in the clinic. Optimization is dependent on being able to monitor the cellular events that take place once the dendritic cells have been injected in vivo, and to establish whether antigen-specific immune responses to the tumour have been induced. Here we describe the use of magnetic resonance imaging (MRI) as a simple, non-invasive approach to evaluate vaccine success. By loading the dendritic cells with highly magnetic iron nanoparticles it is possible to assess whether the injected cells drain to the lymph nodes. It is also possible to establish whether an antigen-specific response is initiated by assessing migration of successive rounds of antigen-loaded dendritic cells; in the face of a successfully primed cytotoxic response, the bulk of antigen-loaded cells are eradicated on-route to the node, whereas cells without antigen can reach the node unchecked. It is also possible to verify the induction of a vaccine-induced response by simply monitoring increases in draining lymph node size as a consequence of vaccine-induced lymphocyte trapping, which is an antigen-specific response that becomes more pronounced with repeated vaccination. Overall, these MRI techniques can provide useful early feedback on vaccination strategies, and could also be used in decision making to select responders from non-responders early in therapy**.**

## Introduction

Dendritic cell (DC)-based vaccines can produce striking remissions of advanced disease [1], [2], but the clinical response rate in most studies is less than 10% [3]. To improve the response rate to DC-based vaccination, there are numerous parameters yet to optimise, such as the process of ex-vivo generation, the antigen loading procedure, selecting the ideal subtype and activation state of the DCs, defining the best route of delivery, and defining the optimal dosing schedule. To investigate these factors, it is necessary to monitor the immune responses generated by vaccination. Although several types of assays exist, typically based on in vitro analysis of induced T cell responses, these procedures have not been applied in a standardised manner and have not been consistently correlated with clinical responses [4]. Furthermore, many DC-based vaccination strategies utilize antigens in the form of whole tumour lysates or tumour-derived nucleic material, so that the precise antigens presented to T cells are not defined, making direct analysis of antigen-specific T cell responses difficult.

Analysis of some parameters of DC-based vaccination may be expedited by development of simple strategies to assess the early cellular interactions required to generate successful immune responses. Following injection by the commonly used routes of delivery (subcutaneous, intradermal or intravenous), the DCs must migrate via the vascular or lymphatic networks to the local lymph nodes to present their antigens to T cells. Indeed, the ability of the injected cells to successfully migrate to the appropriate anatomical location in the lymphoid tissues is likely to be critical factor in vaccination outcome (reviewed in [1]). Simple, non-invasive imaging strategies that can be used to evaluate migration to the lymph nodes could therefore be particularly informative in this setting.

Once located in the lymph nodes, the injected DCs drive T cells with specificity to the presented antigens to proliferate and differentiate into effectors cells. Most assays of vaccine efficacy are reliant on detecting these expanded populations of antigen-specific T cells, which can take weeks to months to occur (often requiring many “booster” injections), with the in vitro assays used to enumerate T cells often beset with problems of sensitivity [4]. However, there are some relatively early events that may be amenable to evaluation by non-invasive strategies. First, animal studies have shown that induction of a potent cytotoxic immune response is accompanied by cytotoxicity directed at the DCs themselves [5], [6]. This is apparent as a reduction in homing of antigen-loaded DCs to the lymph nodes in subsequent rounds of vaccination, as the injected cells can be eliminated on route to the lymphoid tissues. This phenomenon has previously been observed using fluorescent labelling strategies and flow cytometry, but may be amenable to a non-invasive strategy using imaging technology. Second, induction of a T cell response is accompanied by lymphocyte trapping, involving sequestration of circulating lymphocytes into lymphoid tissues peaking 24–48 h after antigen exposure, which was first described over 30 years ago [7]–[11]. Not only is this largely initiated by an antigen-specific process, but it can be associated with 3-fold increases in lymph node size [12], suggesting that monitoring lymph node size could be exploited as a means to evaluate vaccine-induced responses.

Magnetic resonance imaging (MRI) is an imaging tool with high resolution used widely in clinical practice that may be particularly suited to analysis of cell-based therapies. MRI potentially provides both the capacity to assess homing of the injected cells, and the ability to evaluate the architecture and size of the targeted lymphoid tissue. It has previously been shown that DCs labelled with contrast agents comprising of superparamagnetic iron oxide nanoparticles (hereafter referred to as “IONP”) can be successfully detected in draining lymph nodes by *T_2_*-weighted MRI [3], [4], [11]–[19]. The success of this technique was shown to be largely dependent on efficiency of uptake of the nanoparticles by DCs, with the level of contrast enhancement determined by the concentration of magnetic particles within the cells. Uptake can be improved by increasing the concentration of nanoparticles in cell culture or by coating the nanoparticles with antibodies for cell-surface receptors such as CD11c, but this comes at the cost of reduced cell viability [18]. An alternative strategy is to use nanoparticles with stronger magnetic properties, which induce more efficient transverse relaxation of protons and greater *T_2_* contrast enhancement. We recently developed a facile method to produce nanoparticles with an iron core surrounded by an oxide shell, which exhibit superior magnetic properties over fully oxidized particles [20]–[22]. These nanoparticles (hereafter referred to as “Fe NP”) could be dispersed in aqueous solution after coating with a biocompatible ligand di-mercaptosuccinic acid (DMSA), and proved to be excellent contrast agents in MRI [23].

Here we report on the use of MRI with Fe NP as a means to provide useful early feedback on DC-based vaccination strategies.

## Materials and Methods

### Ethics statement

All mice were handled in accordance with the Animal Ethics Policy 2008R7M, approved by the Animal Ethics Committee of Victoria University of Wellington. Mice were not used in experiments until they reached 6 weeks of age. All MRI scans were performed under ketamine/xylazine anaesthesia and all efforts were made to minimize suffering.

### Mice

Breeding pairs of C57BL/6 mice were originally from the Animal Resources Centre, Perth, Australia, and were bred and maintained in the Biomedical Research Unit of the Malaghan Institute of Medical Research.

### In vitro culture media and reagents

All cultures were in complete Iscove’s Minimum Essential Medium (cIMDM) consisting of IMDM supplemented with 5% fetal calf serum (Sigma Aldrich, Auckland, NZ), 2 mM glutamax, 100 U/ml penicillin, 100 µg/ml streptomycin and 50 µM 2-mercaptoethanol (all Invitrogen, Auckland, New Zealand). The murine glioma line GL261 was obtained from DCTD Tumor Repository, National Cancer Institute at Frederick (MD, USA). The murine thymoma E.G7-OVA cells expressing a cDNA encoding the chicken OVA sequence [24] were obtained from American Tissue Type Collection (Manassas, VA, USA) and were maintained in cIMDM supplemented with 0.5 mg/ml geneticin. All cell-lines were regularly tested for mycoplasma by PCR (e-Myco, Intron BioTechnology, Korea). The NKT cell ligand a-galactosylceramide (a-GalCer) was manufactured as described by Lee et al [25] and solubilized in 150 mM NaCl, 0.5% Tween 20. The synthetic peptide OVA_257–264_ (SIINFEKL) was purchased from Chiron Mimotopes (Clayton, Australia).

### Preparation of antigen-loaded DCs

Bone marrow-derived DCs were prepared according to the method of Lutz et al [26] using 20 ng/ml GM-CSF and 20 ng/mL IL-4 prepared in-house. All cultures were supplemented with 100 ng/ml of bacterial lipopolysaccharide (LPS, Sigma-Aldrich) on day 6 to induce maturation. In some experiments, Fe NP or IONP was added to the cultures on day 5 at 5 µg Fe/ml. For antigen loading, in some experiments the culture medium was supplemented with 1 µM OVA_257–264_ for 4 h on day 7, and then the DCs were washed in IMDM before use. In other experiments the medium was supplemented on day 6 with 15 µg/ml of tumour lysate prepared by four cycles of freeze thaw. The lysates contained no viable cells, as determined trypan blue (Invitrogen) exclusion, and protein content was established using a Biorad protein assay kit (BioRad, Auckland, NZ). Where indicated in the text, 200 ng/ml α-galactosylceramide (α-GalCer) was added as to the cultures on day 5 to serve as an immune adjuvant. All cells were harvested on day 7, washed twice with IMDM, and resuspended in phosphate buffered saline (PBS) for injection.

### Injection of DCs

Antigen-loaded DCs (1×10^6^ cells) were injected subcutaneously into the upper hindlimb in final volume of 50 µl PBS. Where indicated in the text, some recipients were primed one week earlier by intravenous injection of DCs loaded with both antigen and α-GalCer (1×10^6^ cells).

### In vivo cytotoxicity assay

The cytotoxic activity induced by vaccination was measured by the VITAL assay [27]. As targets, syngeneic splenocyte populations were loaded separately with 50 nM, 5 nM, or 0.5 nM OVA_257–264_ peptide in complete medium for 2 h. and then labelled with 2 µM, 500 nM or 80 nM carboxy-fluoroscein succinimidyl ester (CFSE: Molecular Probes) for 8 mins at room temperature. Labelling was quenched with fetal calf serum and the cells were washed twice in complete medium and once in IMDM. As a control population, splenocytes without peptide were labelled with 10 µM Cell Tracker Orange, (CTO; Molecular Probes) in complete medium for 15 mins at 37°C, and washed in complete IMDM and twice in IMDM. Equal proportions of all four cell populations were mixed together and 1×10^7^ cells were injected intravenously into groups of immunized and naive mice. Assessment of specific lysis of the peptide-loaded targets relative to controls was assessed by flow cytometry 24 h later in lymph node tissue from euthanized animals.

### Determination of Fe content in labeled DCs

Measurement of iron concentration was by flame atomic absorption spectroscopy on DCs labelled with Fe NP or IONP, with unlabelled DCs used as controls. The cells (1×10^6^) were suspended in 0.5 ml concentrated HCl and diluted up to 3 ml with de-ionized water for analysis, Measurements were made with a GBC 906AA spectrometer (GBC Scientific, Auckland, NZ) using an air-acetylene flame, and then compared to a standard curve generated from iron at known concentrations.

### MRI of labelled DCs dispersed in agar

MRI was performed on DCs that were labelled with Fe NP or IONP, and then dispersed at 100–500 cells/µl into plastic microtubes containing 200 µl of 1% agar (Sigma-Aldrich). MRI was performed using a Bruker Instruments AVANCE400 nuclear magnetic resonance (NMR) spectrometer (Bruker, Alexandria, NSW, Australia) equipped with a Bruker Micro 2.5 imaging module. MR images were acquired at 9.4 T using a 2D multi-slice spin-echo sequence, at room temperature, with the following parameters: echo time (TE)  = 8 ms, repetition time (TR)  = 2000 ms, pixel size  = 100 µm×100 µm, slice thickness  = 0.5 mm, number of echoes  = 64, 4 averages, total experiment time  = 16 minutes. Measurements were repeated on four separate samples at each cell concentration.

### In vivo MRI

Mice were anaesthetized by IP injection with 100 mg/kg ketamine and 10 mg/kg xylazine (both from Phoenix Pharm, Auckland, NZ). Lacrilube (Allergan, Auckland, NZ) was applied to the cornea to prevent dessication. MRI was performed using a clinical 1.5 Tesla MR scanner (Philips Healthcare, North Ryde, NSW, Australia), equipped with a wrist solenoid coil. The mice were positioned in the centre of the wrist coil and the same protocols were used for all images. *T_2_*-weighted multi-slice, multi-echo, spin-echo images were acquired with the following parameters: TE  = 54 ms, TR  = 2000 ms, pixel size  = 300 µm×300 µm, thickness  = 1 mm, number of echoes  = 8, averaged from a total of 3 scans, total experiment time  = 4 minutes 24 seconds. Both an axial and coronal series of images were perfomed. Images were analyzed with ImageJ (National Institutes of Health) by manually selecting a region of interest (ROI) corresponding to the inguinal lymph nodes and integrating the signal intensity of each ROI. The area was measured by tracing around the border of the lymph node and its surrounding fat on a coronal slice. The area of each lymph node was taken as an average from the two coronal slices in which it had the greatest area.

### Fluorescence labelling of DCs

DCs were incubated in 2 µM of CFSE in PBS for 8 mins at room temperature. After incubation, an equal volume of foetal calf serum was added and cells were washed twice in complete medium and once in IMDM before injection into mice.

### Tumour challenge experiments

Groups of mice (*n*  = 5) mice received a subcutaenous injection of 1×10^6^ E.G7-OVA cells into the left flank. Tumour size was calculated by caliper measurements as the product of bisecting tumour diameters. Mice were killed when the tumour size reached 150 mm^2^, or earlier if mice became visibly unwell or if the tumour became ulcerated.

### Analysis of lymph nodes by flow cytometry

The cell types within the inguinal lymph nodes were assessed 24 h after MRI scanning by flow cytometry. The tissue was teased through gauze to provide single cell suspensions which were suspended in staining buffer consisting of PBS containing 1% fetal calf serum (Sigma-Aldrich), 0.01% sodium azide and 2 mM EDTA (both Invitrogen). Non-specific Fc-receptor mediated antibody binding was blocked by incubation with anti-CD16/32 antibody (prepared in house) at 4°C for 10 min, and then washed with staining buffer. The cells were then stained with the indicated antibodies at 4°C for 15 min in staining buffer, and washed twice. Antibodies used were CD4:PerCP, NK1.1:FITC, CD8:V500, CD3:PE-Cy7, CD62L:PE, CD44:APC, B220(CD45R):PETR (all Becton Dickinson, Auckland, NZ); CD8:PacBlue (Biolegend); CD11b:PE, CD11c:APC-Cy7 (eBioscience). To distinguish dead cells, 0.1 µg/ml DAPI (4,6-diamidino-2-phenylindole) or 50 ng/ml of propidium iodide (both BD Pharmingen, Auckland, NZ) was added to the cells before acquisition. Flow cytometry was performed using a BD FACScalibur or BD LSRII SORP (Becton Dickinson) and analysed using FlowJo software (Treestar, Ashland, OR, USA). Compensation was performed with anti-rat and anti-hamster compensation beads (Becton Dickinson). Cell types were defined by the gating strategy seen in Figure S2.

### Statistical methods

The measurements of signal intensity in the lymph nodes on T_2_ weighted MRI showed a non-normal, bimodal distribution. This was observed in all lymph node groups, including control and contralateral nodes without Fe NP or IONP, which may reflect volume averaging effects from water protons in adjacent subcutaneous tissue along the slice selection axis (which was three times as thick as the other two dimensions in order to increase signal to noise ratio). Therefore non-parametric statistical analysis was applied to all T_2_ weighted MR contrast measurements in vivo. The Mann-Whitney rank sum test was applied to two group comparisons with the Kruskall-Wallis test with a Dunn’s post-test applied to multiple group comparisons. For all other results, parametric statistical tests were employed and include unpaired two-tailed t-test for two-group comparisons, log-rank for tumour-free survival analysis and ANOVA with Bonferroni post test for multiple group comparisons. All error bars shown in graphs represent the standard error of the mean. P-values of <0.05 were considered significant. All statistical calculations were produced with Graphpad Prism Version 4 (Graphpad Software Inc, San Diego, CA, USA).

## Results

### Using MRI to detect homing of DCs to lymph nodes

**Figure 1:**
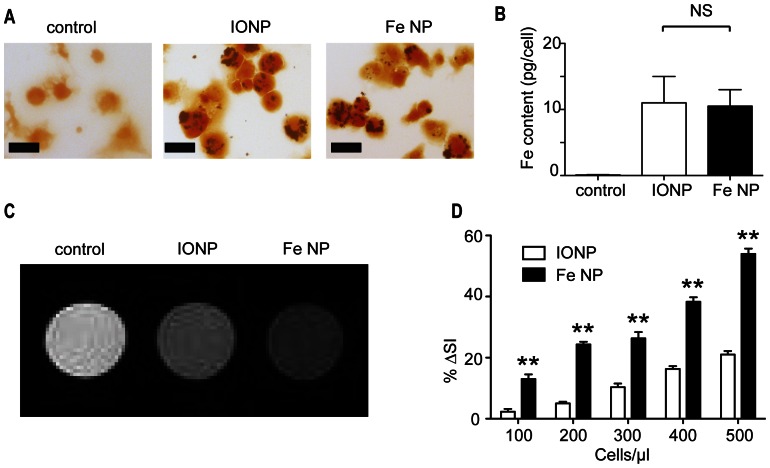
DCs labelled with Fe NP are detected more effectively by MRI than DCs labelled with IONP. (A) BM-DCs were incubated with 5 µg Fe/ml of Fe NP or IONP, or with PBS as a control, and then treated with Perl’s stain to detect uptake of Fe by light microscopy (Scale bar  = 10 µm). (B) Atomic absorption spectroscopy was performed on three separate cultures per treatment group to determine the mean Fe content per cell (± SEM). (C) BM-DCs from the same groups were dispersed at different concentrations in 1% agar and imaged with MRI using a *T_2_*-weighted spin-echo sequence at 9.4 T. Four dispersions were assessed at each concentration. Sample images are shown. (D) The changes in *T_2_*-weighted signal intensity of the Fe NP and IONP groups were compared to the controls and plotted as mean %▵ signal intensity ± SEM, where %▵SI  =  (signal intensity of cell dispersion) - (signal intensity of control)/(signal intensity of control). (**P<0.01).

To investigate the utility of using Fe NP in tracking DCs in vivo, we first assessed their uptake by murine bone marrow-derived DCs (BM-DCs), and compared this to uptake of IONP that had been prepared according to conventional methods [28], and coated with DMSA in the same manner as was used for Fe NP [29]. Analysis of iron levels retained in the cells by light microscopy using Perl’s stain showed no discernible difference in uptake of the different nanoparticles (Fig. 1A). This was confirmed by flame atomic absorption spectrometry, which showed that the amount of iron acquired by DCs from Fe NP and IONP was similar (10 pg Fe per cell) (Fig. 1B). However, when these DCs were dispersed in agar, and subject to MRI at 9.4 T, the cells incubated with Fe NP produced significantly greater change in *T_2_* relaxation in vitro across a range of cell concentrations, with this “negative enhancement” apparent as dark regions on the scans (Fig. 1C, D). From this MRI data, the amount of *T_2_* reduction per cell was calculated as 0.1% per cell for DCs incubated with Fe NP versus 0.05% per cell for those labelled with IO (95% C.I. 0.08–0.11 and 0.04–0.06 respectively).

**Figure 2:**
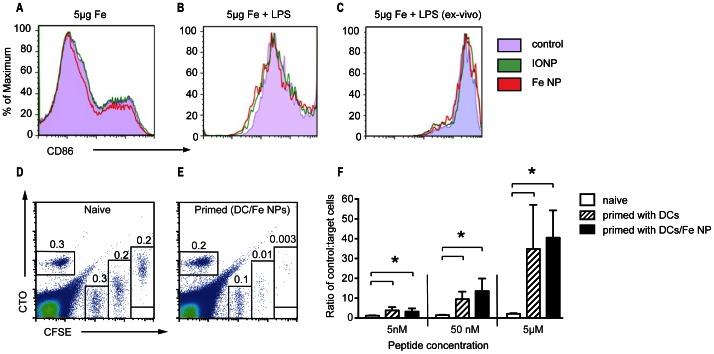
Incubation with Fe NP does not affect DC function. (A, B) The expression of the maturation marker CD86 was assessed by flow cytometry on BM-DC cultures supplemented with 5 µg Fe/ml of Fe NP or IONP, or PBS, (control) on day 5, with or without addition of LPS 24 h later. (C) BM-DCs were supplemented with 5 µg Fe/ml of Fe NP or IONP, or PBS, and injected subcutaneously into mice (*n* = 5). The expression CD86 was assessed by flow cytometry on CD-11c+ DCs harvested from the draining lymph nodes 48 h later. (D–F) Cytotoxic responses induced by vaccination with OVA_257–265_ peptide-loaded DCs with or without Fe NP were measured in vivo against intravenously administered fluorescent targets loaded with OVA_257–265._ Three target populations were administered, each with a different concentration of peptide and different intensity of labelling. Survival of targets was compared a CTO-labelled control population without peptide. Representative flow cytometry plots from a naïve and an immunised animal are shown (D and E), with results from 5 animals per treatment group expressed as a ratio of control:targets in F. (*P<0.05).

We have previously reported that uptake of Fe NP by cells, including dendritic cells, is not associated with loss of cell viability in ranges typically used for cell imaging [20]. To assess the impact of nanoparticle uptake on cell function, we examined the process of LPS-induced DC maturation. Using a typical 5 µg Fe loading dose of Fe NP, no impact on capacity of LPS to induce upregulation of the maturation marker CD86 on the cell surface was observed (Fig. 2A–C). To assess whether Fe NPs interfere with the stimulatory capacity of the DCs, we examined cytotoxic responses induced by vaccination with OVA_257–264_ peptide-loaded DCs with or without Fe NPs. Analysis of elimination of intravenously injected target cells loaded with OVA_257–264_ peptide one week after vaccination showed a similar dose-related cytotoxic response regardless of the presence of Fe NPs. We conclude that DCs can be loaded with Fe NP to provide superior contrast in *T_2_*-weighted MRI without compromise in cell viability or function.

**Figure 3:**
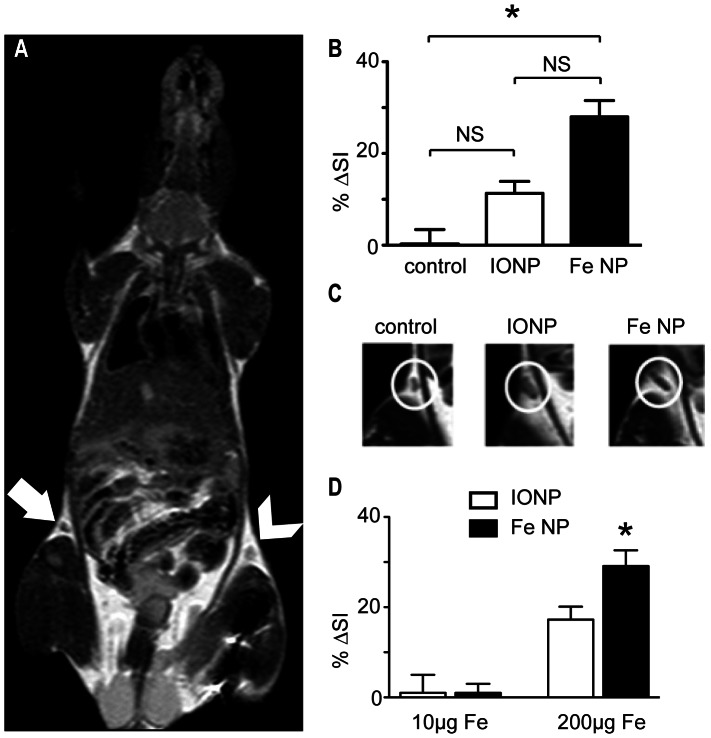
MRI of draining lymph nodes following injection with DCs labelled with Fe NP or IONP. (A) BM-DCs were labelled with Fe NP, IONP or PBS only (control), and injected into the upper hind limbs of different groups of naive mice (*n* = 5). MRI was performed 48 h later at 1.5 T. A representative image from an animal injected with the Fe NP-labelled DCs is shown, with the draining lymph node indicated by an arrow, and the contralateral node by an arrowhead. (B) The mean change in *T_2_*-weighted signal intensity per group was calculated, where %▵SI contrast  =  (signal intensity of contralateral node) - (signal intensity in draining lymph node)/(signal intensity of contralateral node). (C) Representative images of draining lymph node from each group are shown. (D) Groups of mice (*n* = 5) were injected subcutaneously in the upper hind limb with 10µg Fe/ml, or 200 µg Fe/ml, of Fe NP or IONP, or with PBS only as a control. MRI was performed 48 h later at 1.5 T, and the mean change in signal intensity per group calculated as in (B). (*P<0.05, NS *P*>0.05)

The ability to detect homing of BM-DCs to lymph node tissue after injection was then tested in vivo, using a standard clinical 1.5 T MRI scanning device. Scans were obtained 48 h after injection of nanoparticle-loaded cells into the upper hind limb, with signal intensity in the draining inguinal lymph node compared to signal intensity in the contralateral node (Fig. 3). Obvious regions of negative enhancement were seen in the inguinal lymph nodes draining the site of injection, with the lymphoid tissue appearing significantly darker relative to the surrounding fatty tissue than observed in the contralateral nodes of the same individual (Fig. 3A). This contrast enhancement was significantly more pronounced in animals injected with BM-DCs loaded with Fe NP than animals treated similarly with BM-DCs loaded with IONP (Fig. 3B, C). To ensure that the changes in contrast in these experiments were due to migration of the labelled BM-DC, and not simply lymphatic drainage of nanoparticles themselves from the injection site, images were prepared from animals that had been injected with the nanoparticles alone, at a dose equivalent to the loading concentration used on the BM-DC. In addition, some animals received nanoparticles at a dose twenty times that of the typical cell-loading concentration. While some negative enhancement was observed in the draining lymph nodes after 48 h with this higher dose (with Fe NP providing the greater enhancement), no contrast enhancement was observed with either type of nanoparticle injected at the typical loading dose (Fig. 3D). We conclude that the negative enhancement observed in the draining lymph node by MRI after injection of nanoparticle-loaded BM-DCs was a consequence of accumulation of the injected cells at this location. Significantly, this useful non-invasive assay of DC function in vivo was substantially improved by the highly magnetic properties of Fe NP.

### Using MRI to detect induction of a cytotoxic immune response

**Figure 4:**
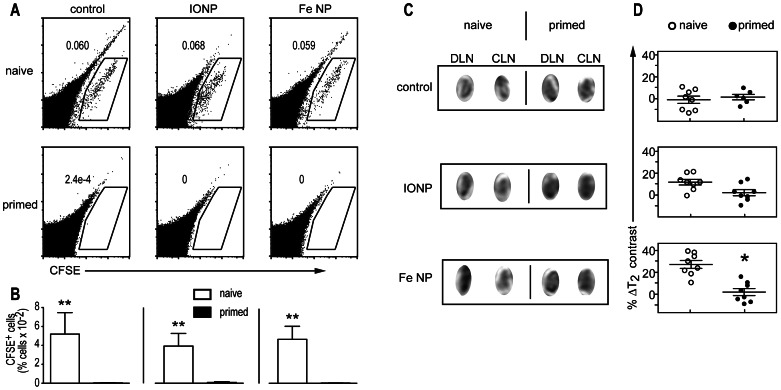
MRI can be used to detect changes in migration of DCs to the draining lymph node in immunised animals. Mice were immunised by intravenous injection with LPS-matured BM-DCs loaded with OVA_257–265_ and α-GalCer (“primed”), or left untreated (“naïve’). One week later, groups of naïve and primed animals (*n* = 5) were injected subcutaneously with LPS-matured BM-DCs loaded with OVA_257–265_ that had been incubated with Fe NP, IONP or PBS (control), and labelled with CFSE. (A) Representative flow cytometry plots of draining lymph nodes 48 h later. (B) Mean number of CFSE^+^ cells detected per group. (C) Representative MRI images of draining and contralateral nodes at 48 h (D) Changes in signal intensity measured by comparing the signal intensity in the draining inguinal lymph node with the contralateral node and plotted as % ▵ signal intensity. Each dot represents an individual comparison. (*P<0.05, **P<0.01).

Successful induction of a potent CD8^+^ T cell response is followed by cytotoxicity directed at antigen-bearing cells, which can include elimination of the DCs responsible for triggering the response in the first place [5]. In the context of DC-based vaccination, this phenomenon is apparent as a severely reduced accumulation of injected cells in the draining lymph nodes once a CTL response has been established [5], [30], which we hypothesized could be observed by imaging technology. To establish a model to investigate this, we first used flow cytometry to assess the accumulation of fluorescent OVA_257–264_ peptide-loaded BM-DCs in draining lymph node tissue in animals primed one week earlier with an intravenous injection of BM-DCs loaded with OVA_257–264_ peptide and the immune adjuvant, α-galactosylceramide (α-GalCer) [31]-[33]. Analysis in primed animals 48 h after injection of the fluorescent peptide-loaded BM-DC showed a consistent and significant reduction in accumulation of fluorescent cells compared to similar analysis in naïve animals (Fig. 4A, B). When the peptide-loaded BM-DCs were also loaded with nanoparticles, there was no impact on the movement of the cells naïve animals, or their susceptibility to elimination in primed animals. We then determined if MRI alone can be used to detect the differences in homing in primed and naive animals (Fig. 4C, D). As negative controls, OVA_257–264_-loaded BM-DCs without nanoparticles were used; no changes in contrast were evident by MRI in the lymph nodes in these animals, as expected. In contrast, when the injected cells were loaded with nanoparticles, negative enhancement was observed in naive animals, but was considerably reduced in primed animals relative to the contralateral lymph node (Fig. 4C). However, only when Fe NP were used did the differences in negative enhancement in naive and primed animals become significant; statistical significance was not reached with IONP (Fig. 4D). In other experiments, BM-DCs that were loaded with Fe NP, but not OVA_257–26_ peptide, were shown to home to the lymph nodes regardless of the presence of a cytotoxic response (not shown). We conclude that it is possible to use assessment of homing of dendritic cells loaded with highly magnetic Fe NP with or without antigen to determine vaccine success in a non-invasive manner by MRI.

**Figure 5:**
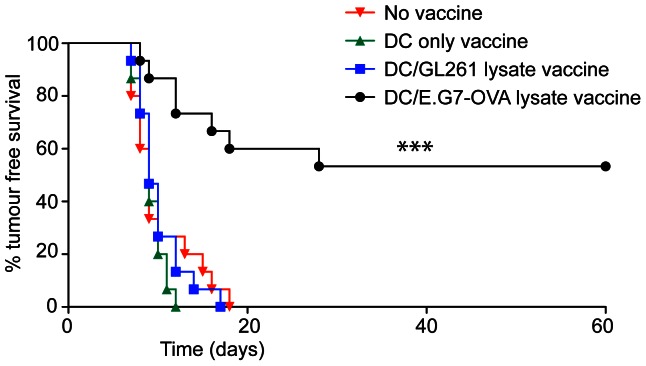
Immunisation with tumour lysate-loaded DCs provides specific protection against tumour challenge. Groups of mice (*n* = 15) were immunised by intravenous injection with LPS-matured BM-DCs alone (DC only), or LPS-matured BM-DCs loaded with either GL261 lysate and α-GalCer, or E.G7-OVA lysate and α-GalCer. One week later these animals, and an additional untreated group, were challenged with 1×10^6^ E.G7-OVA cells in the flank and tumour growth monitored over time. Tumour free survival is for each group is presented. (***P<0.001).

In anti-tumour settings in the clinic it is common to load DC-based vaccines with tumour lysates or tumour RNA, meaning that the antigens targeted are unknown. We have previously reported effective anti-tumour activity in animals with BM-DCs loaded with tumour lysate and α-GalCer [34]. An example is presented in Figure 5, where animals primed by intravenous injection of BM-DCs loaded with α-GalCer and lysate from the murine thymoma cell line E.G7-OVA provided effective protection against subsequent subcutaneous challenge with E.G7-OVA cells. The antigen-specific nature of this response is highlighted by the fact that priming with BM-DC loaded with α-GalCer and lysate from an unrelated murine glioma cell line GL261 did not provide protection against tumour challenge. To determine whether such successfully primed E.G7-OVA-specific responses can be detected by MRI, we assessed whether homing of Fe NP-labelled BM-DCs bearing E.G7-OVA lysate was altered (Fig. S1). Unlike the experiments above, where peptide-specific cytotoxic responses eliminated peptide-loaded BM-DCs on route to the draining lymph nodes, no impact on homing was observed for DC loaded with E.G7-OVA lysate, despite the fact that primed animals could induce specific anti-tumour responses. Therefore, while MRI can be used to observe elimination of antigen-loaded DCs in the face of a potent cytotoxic response, this may not be the most relevant readout in some anti-tumour vaccination settings.

### Using MRI to detect induction of an immune response; assessment of lymphocyte trapping

**Figure 6:**
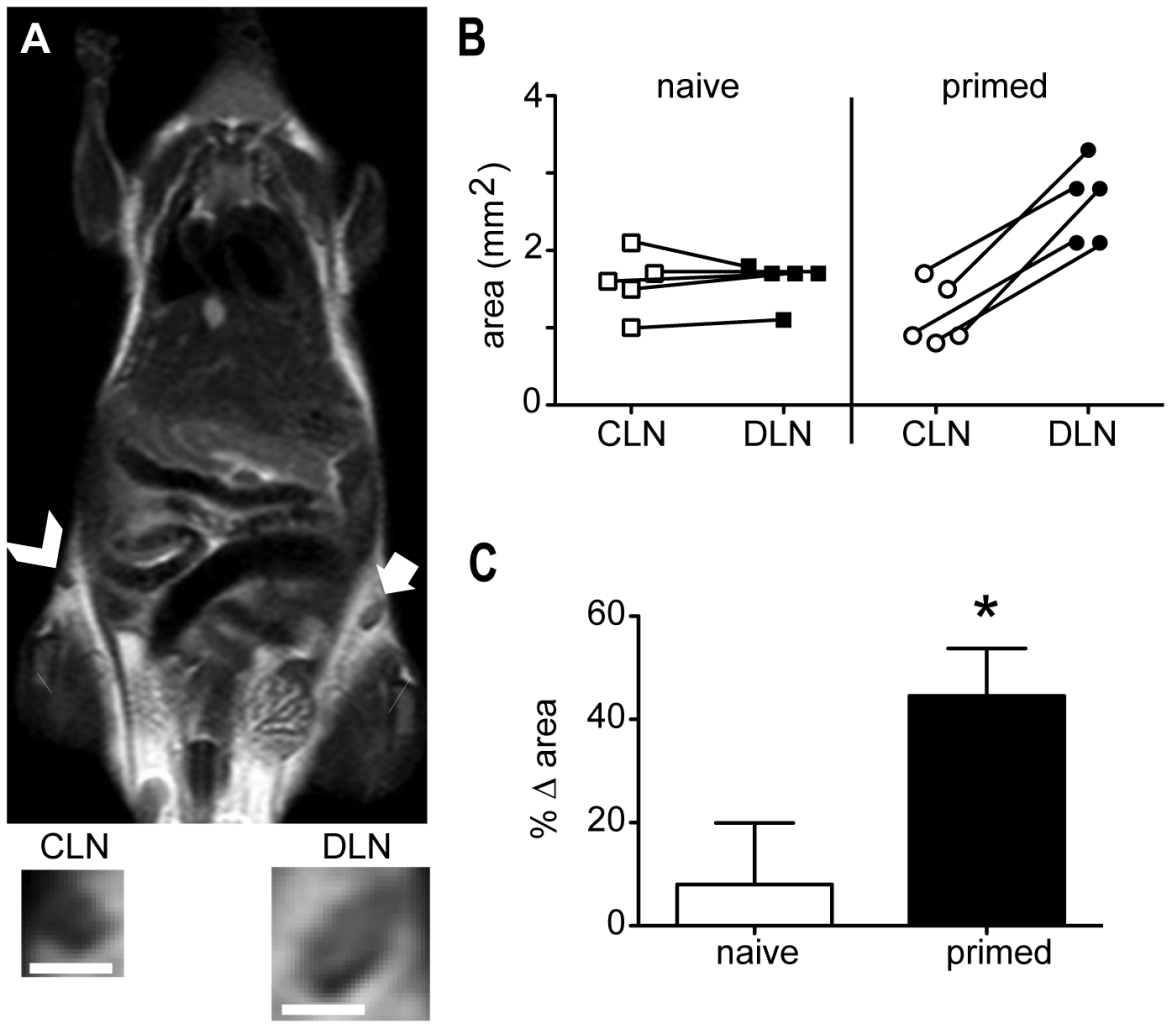
MRI can be used to detect changes in size of the draining lymph node in immunised animals. MRI was used to examine lymph node size in mice treated as in Figure 4. (A) The draining lymph node (arrow and DLN inset) and contralateral node (arrowhead and CLN inset) from a representative of the “primed” is shown. Scale bar  = 1 mm. (B) Graphs showing changes in lymph node area between paired draining and contralateral lymph nodes (*n* = 5). (C) Graph showing the mean percentage difference in the area of the draining versus the contralateral lymph nodes for primed and naive groups, where %▵ area  =  (area of draining lymph node) - (area of contralateral lymph node)/ (area of contralateral lymph node). (*P<0.05).

While successful lysate-based vaccination strategies did not alter homing of subsequent rounds of lysate-loaded dendritic cells, analysis of the MRI images generated did suggest that overall lymph node size was correlated with vaccine outcome. Given that the lymph node enlargement is thought to be driven initially by an antigen-specific process [10], [11], it is not unreasonable to expect that the most measurable increases in draining lymph node size would be observed shortly after a second round of injection with the same vaccine; the numerically enhanced pool of antigen-specific cells in this situation would be capable of orchestrating a much more rapid and profound trapping process. We therefore examined lymph node size in mice first primed intravenously with BM-DCs loaded with OVA_257–264_ peptide and α-GalCer, and then injected subcutaneously seven days later with Fe NP-labelled BM-DCs loaded with OVA_257–264_ peptide. Control animals were only given the subcutaneous injection only. The inguinal lymph nodes draining the subcutaneous injection site, and contralateral nodes, were imaged by MR 30 h later to assess lymph node size (Fig. 6). The areas of the lymph nodes can be compared by plotting the area of each lymph node (Fig. 6B) or by expressing this as a percentage of the area of the contralateral lymph node for each mouse (Fig. 6C). The latter method removes some of the background statistical noise produced by comparing mice of different sizes (up to a 10% difference in mass was observed in 6 week old syngeneic littermates) and obviates the need to use paired statistical tests when comparing groups of mice. Using this form of analysis, a significant increase in the size of the draining lymph nodes was observed in primed animals, but not in naive mice. This response was reproducible, with all of the draining lymph nodes in the primed group being larger than the contralateral nodes in the same mouse.

**Figure 7:**
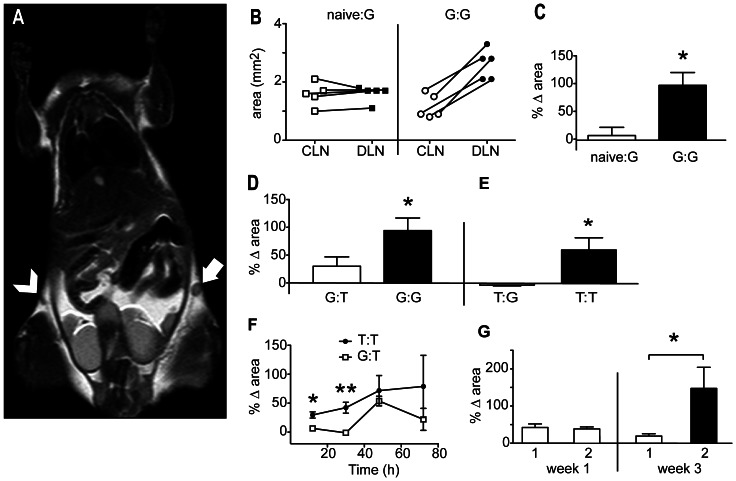
MRI can be used to detect antigen-dependent increases in lymph node size with repeated immunization. LPS-matured BM-DCs loaded with GL261 glioma lysate were injected subcutaneously into naïve animals (naive:G), or mice that were primed intravenously with the same vaccine one week earlier (G:G). MRI was performed 30 h after this “challenge” vaccine. (A) Image showing the draining lymph node (solid arrow) and contralateral node (arrowhead) from a representative from the G:G group. (B) Graphs showing changes in lymph node area between paired draining and contralateral lymph nodes. (C) Graph showing the mean percentage difference in the area of the draining versus the contralateral lymph nodes for each treatment group (*n* = 5). In (D), all mice were primed with the GL261 lysate vaccine described in *A*, then challenged with the same vaccine (G:G), or with a DC-based vaccine prepared from E.G7-OVA thymoma cells (G:T). Mean percent changes in lymph node area per treatment group are shown. (E) The reverse experiment is shown, where animals were first primed with the vaccine prepared from E.G7-OVA cells, and challenged with the same vaccine (T:T) or the GL261 lysate vaccine (T:G). (F) Images were analysed at the indicated times after challenge vaccine in the T:T and G:T groups, and mean percent changes in lymph node area per group plotted (G) All mice received two doses of the same vaccine prepared from E.G7-OVA thymoma cells, one week apart. The mice were then divided into two groups (1 and 2), and MRI performed on all mice after 30 h. Mean percent changes in lymph node area per group are presented as “week 1”. Two weeks later, one group received a third vaccine (black) and MRI was performed on all mice 30 h later. Mean percent changes in lymph node area per group are presented as “week 3”. (*P<0.05, **P<0.01).

A similar experiment was then conducted using DCs loaded with tumour lysate. Responses were first primed by intravenous administration of BM-DCs loaded with E.G7-OVA lysate and α-GalCer, and then the animals were challenged subcutaneously with BM-DCs loaded with E.G7-OVA lysate seven days later. Control animals received the subcutaneous challenge vaccine only. Analysis by MRI 30 h after the subcutaneous vaccine showed a significant increase in size of the draining lymph nodes compared with the contralateral lymph nodes in mice primed with lysate-loaded DCs, whereas no measurable differences were observed when challenge vaccines were administered to naive controls (Fig. 7A–C). These initial results were promising but it remained possible that the enlargement was due to an immune response directed at non-tumour constituents of the vaccine, such as the foetal calf serum in which the BM-DCs were cultured, or the immune adjuvant used. To examine this, mice were primed and challenged with BM-DCs loaded with different lysates; mice initially primed with BM-DCs loaded with GL261 glioma cell lysate were challenged with BM-DCs loaded with GL261 lysate (referred to as the “G:G” group), or E.G7-OVA thymoma lysate (“G:T” group), while the reverse experiments were also conducted (“T:T” and “T:G” groups). Analysis 30 h after vaccine challenge showed that significant increases in draining lymph node size were observed only in animals that had received two rounds of BM-DCs bearing the same lysate (Fig. 7D, E). Thus, the major contribution to enlargement of the draining lymph nodes was a specific immune response to the lysate constituent of the vaccine.

Analysis of a time course of vaccine-induced lymph node enlargement showed that the lysate-specific response remained significant only up to 48 h after the challenge vaccine (Fig. 7F); at later times the analysis was confounded by a general, but somewhat variable, increase in size that is possibly related proliferation of endothelial cells [35].

It was then investigated whether draining lymph enlargement would be a feature of successful re-stimulation after several injections of the same vaccine. Two groups of mice received two injections of glioma lysate-loaded DC vaccines, given subcutaneously one week apart. Lymph node size was measured by MRI 30 h after the second vaccine. Then two weeks later, one group received a third vaccine (the “re-challenge” group); all mice were then imaged again 30 h later. Enlargement of the draining lymph node was seen in all animals 30 h after the second vaccination, as was expected (Fig. 7G). However, the mice in the re-challenge group developed an even more profound enlargement after the third vaccine. In contrast, over the same timeframe (two weeks), the draining lymph nodes of the mice that did not receive this third vaccine were reduced in size to the point that they were no longer significantly larger than contralateral nodes. This timeframe of enlargement followed by reduction in size would therefore make it possible to use draining lymph node size as indicator of success or failure of a boosting strategy with repeated vaccination.

### Cell types involved in lymph node enlargement

**Figure 8:**
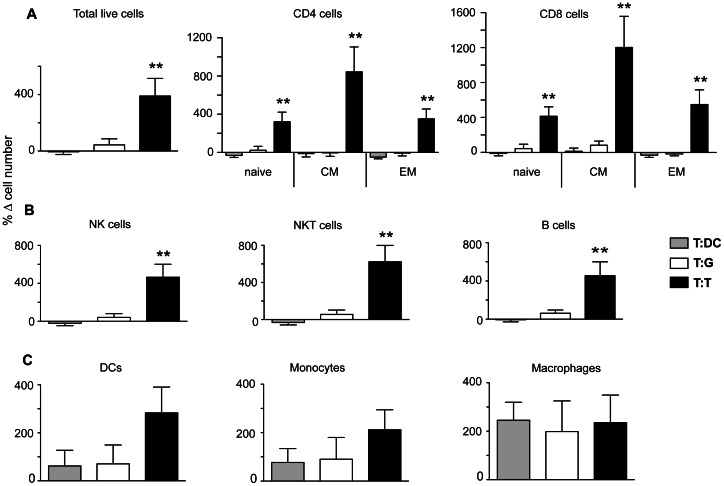
Cell types involved in lymph node infiltration. The draining and contralateral lymph nodes were removed 30 h post-vaccination from groups of mice (*n* = 5*)* that were primed with a DC-based vaccine prepared E.G7-OVA thymoma lysate, and boosted with either DCs only (T:DC), a DC-based GL261 lysate vaccine (T:G) or the original DC-based E.G7-OVA lysate vaccine (T:T). The different cell types indicated were enumerated by flow cytometry, using the gating strategy provided in Figure S2. The increase in each cell-type is plotted as the mean percent difference in cell number, where %Δ cell number  =  (number of cells in DLN) - (number of cells in the contralateral lymph node)/(number of cells in the contralateral lymph node). (*P<0.05, **P<0.01).

To examine the cellular make-up of the lymph nodes when vaccine-specific enlargement was most significant, the draining and contralateral nodes were removed 30 h after the second of two rounds of vaccination with lysate-loaded vaccines, and processed into single cell suspensions for analysis by flow cytometry (Figure 8). Analysis of mice that had been primed with E.G7-OVA thymoma lysate-loaded DCs, and then challenged with the same vaccine (T:T), showed that, on average, there were 400% more cells in the draining lymph nodes compared to the contralateral lymph nodes. This increase in cell numbers alone was of sufficient magnitude to explain the enlargement observed on MRI without involving other mechanisms such as oedema or an increase in vascularity of the lymph node, although these other mechanisms could not be ruled out. The cellular make up of the draining lymph nodes of the T:T group was assessed relative to the contralateral lymph node of the same animals (Fig. 8), and also compared to similar analysis in animals that had been primed with thymoma lysate-loaded DCs and challenged with either GL261 glioma lysate-loaded DCs (T:G), or DCs only (T:DC). The highly significant increase in cellularity in the T:T group was made up of a broad variety of lymphocytes including CD4^+^ and CD8^+^ T cells, B cells, NK cells and NKT cells. Interestingly, there was a trend for the numbers of myeloid cell types such as DCs, monocytes and macrophages, to increase with vaccine challenge regardless of the type of lysate (or whether there was a lysate at all), although this did not reach significance. This may suggest that myeloid cells do accumulate as a consequence DC-based vaccination, but that this is not driven in an antigen-specific manner.

The T cells were further separated into naive, central memory (T_cm_) and effector memory (T_em_) cells [36]. While all T cell numbers were increased in the draining lymph nodes of the T:T group relative to contralateral nodes, the fold-increases in CD4^+^ and CD8^+^ T_cm_ were approximately twice the magnitude of the total cellular increases, suggesting a selective recruitment. We conclude that the vaccine-induced enlargement of the draining lymph node is the result of a broad-spectrum recruitment of lymphocytes, with some bias towards recruitment of T_cm_ cells.

## Discussion

Dendritic cell-based cancer immunotherapy is typically delivered in the form of enriched populations of DC pulsed with defined tumour-associated antigen(s), or pulsed with tumour-derived material where the specific antigens targeted are not known. Using defined tumour-associated antigens has the advantage of stimulating responses with high selectivity for the tumour, limiting the risk of autoimmunity, and also permiting incorporation of high doses of antigen into the vaccine to help induce potent responses. Importantly, with the targeted antigens defined, it easier to monitor induced T cell responses with antigen-based technologies such as ELISpot, or flow cytometry with fluorescent mutimeric peptide/MHC complexes. Vaccines derived from tumour material, in contrast, require no prior knowledge of antigen expression or MHC phenotype, but high quality monitoring is made difficult by not knowing the sequences targeted. However, there is some evidence that the broad immunity provided by this form of vaccine is more clinically effective [37], as responses can be generated to multiple targets, some of which may be patient-specific. There therefore remains significant impetus to develop this type of therapy despite the difficulty in monitoring.

Here we describe the use of MRI as a non-invasive approach to evaluate vaccine success in these different settings. We first evaluated Fe NP as contrast agents to monitor homing of injected DC-based vaccines to draining lymph nodes, the first step required in inducing an immune response. While there have been previous reports of using IONP to provide negative enhancement to distinguish injected DCs from surrounding lymphoid tissue by by MRI, most of these earlier studies have been conducted with high field MRI devices (4.7–11.7 T) [3], [4], [11], [13]–[19]. We reasoned that the higher magnetic properties of Fe NP would allow useful information to be collected on a MRI scanner with a field strength of 1.5 T, which is widely used in clinical practice. In fact Fe NP proved to be very useful contrast agents in this context, with significantly better discrimination of injected DCs in the draining lymph nodes than when IONP were used.

Using this sensitive imaging strategy we tested whether MRI could be used to observe differences between naïve and immunized animals by focusing on DC homing, and lymph node structure, in nodes draining the injection site. In animals that had been successfully primed to elicit cytotoxic responses to MHC class I-binding peptide, we observed severely compromised homing of any further injections of Fe NP-loaded DCs bearing the same peptide. This was not unexpected, as the peptide-specific CD8^+^ T cells that were activated in the priming process are known to have the capacity to eliminate DCs in vivo in an antigen-specific and perforin-dependent manner [6]. These same responses have been commonly observed to provoke strong anti-tumour activity. Despite the reduced homing on repeated vaccine administration, an obvious enlargement of the draining lymph node was observed by MRI within 48 h of secondary rounds of vaccination. It is likely that this reflects early lymphocyte trapping as a consequence of some residual peptide-bearing DCs reaching the lymphoid tissue to restimulate antigen-experienced T cells, with these cells in turn initiating the cascade of molecular signals required to inhibit lymphocyte egress from the node. Combining these observations, it is therefore possible to use MRI to establish whether vaccine-induced priming of a cytotoxic responses to an MHC class I-binding peptide has been successful by comparing images of the draining lymph nodes within 48 h of the first and second rounds of vaccination with Fe NP-loaded DCs. After the first round, successful homing of the DCs will be apparent as a change in negative contrast, but the node will not be obviously enlarged. After the second round, if priming has been successful, homing of DCs will be reduced but the node will be substantially larger. Assessment of non-draining lymph could serve as a control for this technique, with no DC homing or antigen-specific lymph node enlargement expected at this site. Such an assay of draining lymph node tissue may prove to be of more relevance than blood-based assays, as vaccine-induced antigen-specific CD8^+^ T cells have previously been observed in biopsies of the draining lymph node while being largely undetectable in the blood of the same patients [38]. These factors, combined with the non-invasive nature of the assay, warrant consideration of Fe NP-enhanced MRI of lymph nodes to be applied to peptide-based vaccine trials.

When we assessed the capacity to use MRI to assess responses to DC vaccines loaded with whole tumour lysate, we were unable to detect DC elimination after repeated administration. This was despite the fact that the lysate-loaded vaccines were capable of inducing immune activity that retarded growth of subcutaneous tumours, and provoked significant early lymphocyte enlargement on repeated vaccination. Both of these responses were antigen-specific, as lysates from an unrelated tumour could not induce the same activity. Peptide-loaded DCs are expected to induce a highly focussed response, potentially resulting a large cohort of cytotoxic cells capable of interacting directly with “booster” DCs through recognition of peptide at high density on the cell surface. Lysate-loaded DCs, on the other hand, will produce a broader response, with the target antigens likely to be at much lower density on the DC. Although this broad response is capable of resisting tumour growth, suggesting antigen density on DCs is sufficient to induce an immune response (and the density on tumours is sufficient to make them susceptible to immune attack), it is perhaps surprising that the DCs are not susceptible to the T cells induced, particularly as it is generally thought that higher densities are required for priming than killing. A possible explanation is that DCs are inherently resistant to elimination. It has been observed that DCs can upregulate the serine protease inhibitor (SPI)-6 [39], particularly in mature cells, lending some weight to this possibility. Because expression of SPI-6 has been shown to be incapable of preventing elimination by a peptide-specific response in vivo [40], this protective effect may be overwhelmed in the face of a particularly potent cytotoxic response.

It has been demonstrated that antigen-specific CD4^+^ T cells can protect DCs from CTL-mediated elimination through binding to CD40 on DCs [41]. Indeed, MHC class II-negative DCs show reduced survival in vivo [42], presumably reflecting an inability to receive this, or other, protective signals. A major difference between peptide-loaded and lysate-loaded DCs in our assays may therefore be the ability to induce activation of CD4^+^ T cells. The DC culturing systems used in both situations involve fetal bovine serum, which will contribute some MHC class II epitopes, but the lysates are likely to contain a number of additional tumour-specific MHC class II epitopes. In fact, in some models, the anti-tumour activity of lysate-based vaccines is CD4^+^ T cell mediated [43], [44], including our studies of a glioma vaccine similar to the one used here [45], suggesting there may be a significant cohort of CD4^+^ T cells capable of providing protection against DC elimination. It should also be noted that while lysate-loaded DC do induce T cells with anti-tumour activity, it is possible that this function uses quite different effector activities to those required for DC elimination. In some animal models of tumour immunotherapy, release of cytokines such as IFN-γ plays an important antitumour role [46], [47], perhaps through their anti-angiogenic qualities, whereas the killing of DCs in the face of a potent peptide-induced response has been shown to be strictly dependent on expression of perforin [6].

In spite of the absence of DC elimination using lysate-loaded DCs, it is still possible to use an MRI-based analysis of lymph node enlargement as a readout of vaccine success. Within the first 48 h of administration of lysate-loaded DCs into a previously immunized animal this enlargement is driven by an antigen-specific process, resulting in a broad-spectrum recruitment of lymphocytes with some bias towards recruitment of T_cm_ cells. Beyond 48 h, proliferation of endothelial cells can increase lymph node cellularity, forming an expanded network of blood vessels that increases the supply of cells entering the node [35]. Of particular interest, the early enlargement of draining lymph nodes became more pronounced with further vaccination, meaning that interpretation of MRI images can be validated with each subsequent round.

We conclude that MRI can be used to detect crucial early events in the generation of immune responses after dendritic cell-based vaccination. The use of highly magnetic Fe NP will improve analysis of DC homing to the draining lymph node, and depending on the antigen-loading strategy used, can potentially be used to assess DC elimination as a consequence of vaccine-induced responses. Assessing lymph node enlargement at the appropriate time will provide another readout of vaccine success, a response that is amplified on repeated rounds of vaccination.

## Supporting Information

Figure S1 **Vaccination with DC/lysate fails to abrogate DC migration to the draining lymph node.** Mice were immunised by intravenous injection with 1×10^6^ LPS-matured BM-DCs loaded with 35 µg of GL-261 tumour cell lysate and α-GalCer (“primed”), or left untreated (“naïve’). One week later, groups (*n* = 5) of naïve and primed animals were injected subcutaneously with 1×10^6^ LPS-matured BM-DCs loaded with 35 µg of GL-261 that had been incubated with PBS (control), IONP or Fe NP and labelled with CFSE. (A) Representative flow cytometry plots of draining lymph nodes 48 h later. (B) Mean number of CFSE+ cells detected per group. (C) Histogram plots of the number of CFSE+ cells recovered from the lymph nodes are shown for different groups based on the amount of GL-261 tumour lysate added to DC culture. (D) Representative MRI images of the draining and contralateral nodes from a member of the Fe NP group, 48 h post injection. (E) Changes in signal intensity measured by comparing the signal intensity in the draining inguinal lymph node with the contralateral node and plotted as percentage change in *T_2_* weighted signal intensity (% ΔSI). Each dot represents an individual comparison.
(TIF)

Figure S2 **Analysis of cell types in lymph nodes by flow cytometry.** Cells harvested from lymph nodes were stained with antibodies and defined by the gating strategy depicted. The gates were set using flow data from the contralateral lymph nodes of the DC only group as these were the closest to a true naive cell population. All cells went through gates to remove clumped cells, debris, and a DAPI negative gate to remove dead cells (top row). Different cell types were then gated in the following way: CD4^+^ T cells (B220^−^/CD4^+^), CD8^+^ T cells (B220^−^/CD4^+^), B cells (B220^+^), NK cells (B220^−^/CD4^−^/CD3^−^/NK1.1^+^), NKT cells (B220^−^/CD3^+^/NK1.1^+^), DCs (B220^−^/CD11c^+^), monocytes (B220^−^/CD11c^−^/Ly6^Hi^) and macrophages (B220^−^/CD11c^−^/F480^+^).
(TIF)
