# Supplementary figures and images for: Using Magnetic Resonance Imaging to Evaluate Dendritic Cell-Based Vaccination
Source: PLoS One. 2013 May 29;8(5):e65318. doi: 10.1371/journal.pone.0065318 (PMC3667033; doi:10.1371/journal.pone.0065318)

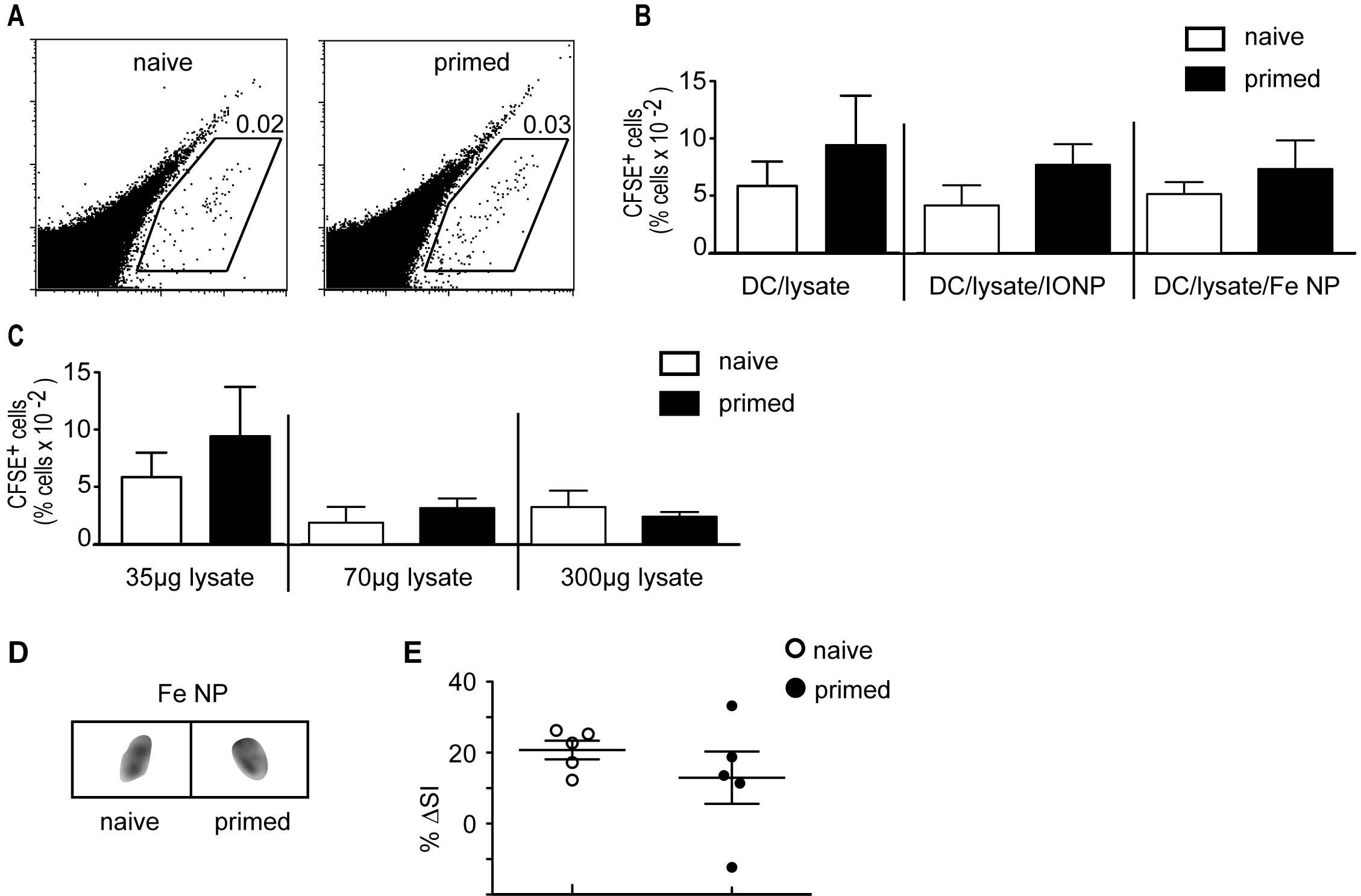

Supplement: Figure S1 — Vaccination with DC/lysate fails to abrogate DC migration to the draining lymph node. Mice were immunised by intravenous injection with 1×10^6^ LPS-matured BM-DCs loaded with 35 µg of GL-261 tumour cell lysate and α-GalCer (“primed”), or left untreated (“naïve’). One week later, groups (n = 5) of naïve and primed animals were injected subcutaneously with 1×10^6^ LPS-matured BM-DCs loaded with 35 µg of GL-261 that had been incubated with PBS (control), IONP or Fe NP and labelled with CFSE. (A) Representative flow cytometry plots of draining lymph nodes 48 h later. (B) Mean number of CFSE+ cells detected per group. (C) Histogram plots of the number of CFSE+ cells recovered from the lymph nodes are shown for different groups based on the amount of GL-261 tumour lysate added to DC culture. (D) Representative MRI images of the draining and contralateral nodes from a member of the Fe NP group, 48 h post injection. (E) Changes in signal intensity measured by comparing the signal intensity in the draining inguinal lymph node with the contralateral node and plotted as percentage change in T2 weighted signal intensity (% ΔSI). Each dot represents an individual comparison. (TIF) [file pone.0065318.s001.tif]

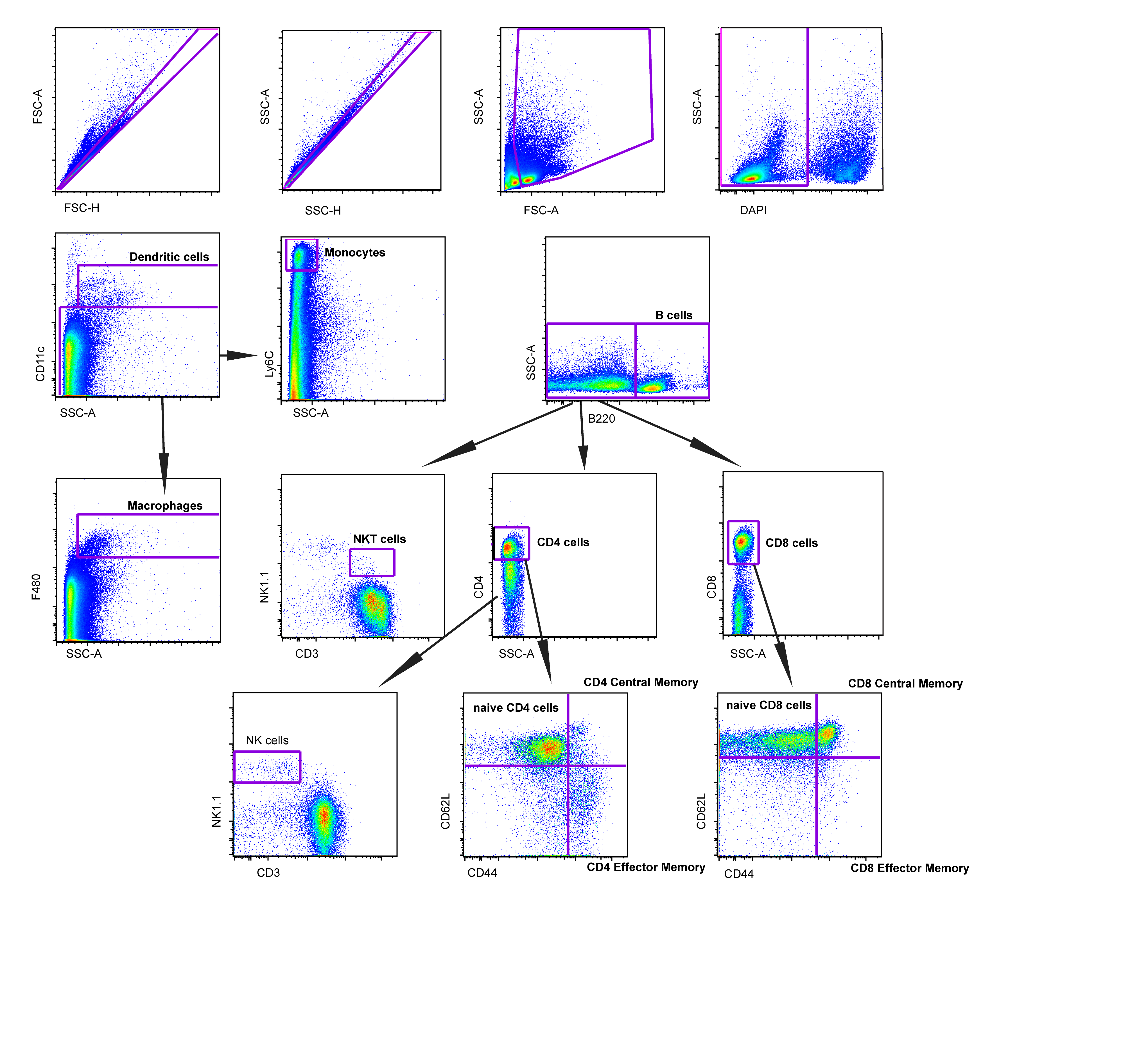

Supplement: Figure S2 — Analysis of cell types in lymph nodes by flow cytometry. Cells harvested from lymph nodes were stained with antibodies and defined by the gating strategy depicted. The gates were set using flow data from the contralateral lymph nodes of the DC only group as these were the closest to a true naive cell population. All cells went through gates to remove clumped cells, debris, and a DAPI negative gate to remove dead cells (top row). Different cell types were then gated in the following way: CD4+ T cells (B220−/CD4+), CD8+ T cells (B220−/CD4+), B cells (B220+), NK cells (B220−/CD4−/CD3−/NK1.1+), NKT cells (B220−/CD3+/NK1.1+), DCs (B220−/CD11c+), monocytes (B220−/CD11c−/Ly6Hi) and macrophages (B220−/CD11c−/F480+). (TIF) [file pone.0065318.s002.tif]
